# Mid-life psychosocial work environment as a predictor of work exit by age 50

**DOI:** 10.1371/journal.pone.0195495

**Published:** 2018-04-05

**Authors:** Stephen A. Stansfeld, Ewan Carr, Melanie Smuk, Charlotte Clark, Emily Murray, Nicola Shelton, Jenny Head

**Affiliations:** 1 Centre for Psychiatry, Wolfson Institute of Preventive Medicine, Barts and the London School of Medicine and Dentistry, Queen Mary University of London, London, United Kingdom; 2 Department of Epidemiology and Public Health, University College London, London, United Kingdom; CUNY, UNITED STATES

## Abstract

**Objectives:**

To examine whether psychosocial work characteristics at age 45 years predict exit from the labour market by the age of 50 years in data from the 1958 British Birth Cohort.

**Methods:**

Psychosocial work characteristics (decision latitude, job demands, job strain and work social support at 45 years and job insecurity at 42 years) measured by questionnaire were linked to employment outcomes (unemployment, retirement, permanent sickness, homemaking) at 50 years in 6510 male and female participants.

**Results:**

Low decision latitude (RR = 2.01, 95%CI 1.06,3.79), low work social support (RR = 1.96, 95%CI 1.12,3.44), and high job insecurity (RR = 2.27, 95%CI 1.41, 3.67) predicted unemployment at 50, adjusting for sex, housing tenure, socioeconomic status, marital status, and education. High demands were associated with lower risk of unemployment (RR = 0.50, 95%CI 0.29,0.88) but higher risk of permanent sickness (RR = 2.14, 95%CI 1.09,4.21).

**Conclusions:**

Keeping people in the workforce beyond 50 years may contribute to both personal and national prosperity. Employers may wish to improve working conditions for older workers, in particular, increase control over work, increase support and reduce demands to retain older employees in the workforce.

## Introduction

In the context of an ageing population, accompanied by improved health at older ages, there is an increasing policy focus on retaining employees in the workforce. This applies to both retaining employees beyond conventional retirement ages but also identifying why employees drop out of the workforce at younger ages. While many studies have examined determinants of workforce exit around statutory retirement age (60+), less is written about earlier exit from the workforce by the age 50 which is the subject of this paper.

One aspect of working lives that may either hasten employees’ work exit or encourage extended working beyond statutory retirement age is their psychosocial work environment. Employees may leave the workforce for a variety of reasons including early retirement, unemployment or for health-related reasons (permanent sickness or disability pension), and these outcomes have previously been linked to adverse psychosocial work characteristics. Low decision latitude, that is low control over work and little opportunity for use of skills, predicts earlier retirement [1–4] and higher levels of control at work are related to delayed retirement [5]. Psychological demands, in terms of fast work pace and conflicts in priority between work tasks, in general, do not predict early retirement[1,3,6] except in some occupational groups (e.g. nurses, Jensen et al [7]) while there is some evidence that low support from managers predicts intention to retire early [8] or early retirement [9]. Job insecurity is also associated with early retirement [10,11].

Psychosocial work characteristics also influence risk of sickness absence. Decreases in decision latitude and increases in job demands predict long spells of sickness absence [12]. Role conflict, low reward, and poor management quality predict long-term sickness absence in women and emotional demands predict long-term sickness absence in men [13]. Recurrent spells of absence in turn predict permanent sickness-related absence from the workforce [14,15]. Sickness absence rates also vary by occupation [16], which in turn, is associated with psychosocial work characteristics.

Most previous studies of the association of psychosocial work characteristics with health-related work exit have been conducted in countries where disability pension is awarded. Disability pension is not awarded by the state in the UK; people with permanent sickness which prevented them from finding jobs were eligible to apply for incapacity benefit until 2007–8. High job strain predicts increased risk of disability pension [17], low job control predicts disability retirement in women [18] and low decision authority and low variety at work predict disability pension in both men and women [19]. A combination of demands and low quality of leadership also predicts disability pension [20]. Similarly, high demand is related to increased risk of disability in a large sample of Swedish twins while higher levels of control is related to lower risk of disability pension award [21]. Interventions to increase decision making and social support at work reduce short spells of absence but it is not clear how they affect permanent sickness and disability [22] although low social support at work and low job security have been shown to predict disability pension in women [23].

Low job control [2, 24], low job satisfaction [25] and job demands predict unemployment [26]. Mental ill-health predicts unemployment and the quality of working conditions influences whether people with mental ill-health receive disability benefits [27].

We examined whether psychosocial work stressors affected these outcomes for workforce exit at 50 years. We also investigated whether these associations might be moderated by social position, sex, and longstanding illness. We hypothesised that permanent sickness, unemployment and early retirement at 50 years would be associated with low decision latitude, low work support and high job insecurity. Permanent sickness in this study is equivalent to not being able to work because of permanent illness and disability. We also hypothesised that the associations of adverse work characteristics and workforce exit would be stronger for people in less advantaged social position and for women. The novel aspects of this study are the opportunity to study workforce exit at an earlier age than most studies, to adjust for several measures of social position and to examine a range of outcomes simultaneously. This paper analyses data from the 1958 British Birth Cohort using data on psychosocial work characteristics at age 45 years to predict exit from the labour market by the age of 50.

## Materials and methods

### Study population

The 1958 Birth Cohort commenced as a perinatal mortality survey that included 98% of all births in England, Scotland and Wales during a week in March 1958 [28]. The cohort members have been followed up and interviewed at ages 7, 11, 16, 23, 33, 42 with a biomedical follow-up at age 45 and further follow up at 50 and 55 years. During the childhood surveys the sample was augmented by immigrants to the UK who were born in the study week giving a total sample of 18,558 participants. Data were obtained from parents and schools (teachers and doctors) on participants at ages 7, 11 and 16 years and through personal interviews at ages 23, 33, 42, 50 and 55 years. At age 33, 11,405 participants, and at age 42, 11,419 participants were interviewed [28]. After exclusions for death, emigration, permanent refusal, armed forces and long-term non-contacts, the 11,971 participants who were still in contact with the study at age 45, were invited to a nurse-led biomedical assessment including measurement of respiratory function, eyesight, hearing and a computer assisted personal interview. The achieved sample was 9,377 with a response rate of 72% of the contacted sample, representing 59% of the eligible sample. At age 50 the achieved sample was 9,790 with a response rate of 80.4% of the contacted sample. The sample included 6510 people in paid work at age 45, based on self-reported labour market status.

Ethical approval for the biomedical survey was given by the South East Multi-Centre Research Ethics Committee (MREC).

### Assessment of work characteristics

Karasek’s job strain model has two dimensions: decision latitude (comprised of decision authority and skill discretion) and psychological demands [29]. Work social support was added as a third dimension to the model [30]. In this study, at 45 years, decision latitude was measured by 6 items, three on skill discretion and three on decision authority. Psychological demands were measured by four items on work pace and conflicting demands; work social support by three items on support from colleagues and supervisors in a self-completion questionnaire. These items were derived from the Whitehall II Study questionnaire [31] version of Karasek’s Job Content Instrument [29]. There was good reliability for each of the subscales: Cronbach’s alpha was 0.79 for decision latitude, 0.66 for psychological demands and 0.81 for work social support. Scores on these scales were divided into tertiles for analyses. Job strain is the combination of high demands and low decision latitude; the job strain category was compared with a composite of all other categories (low strain, active and passive jobs) that constituted low strain. Job insecurity was measured by a 4-point scale: ‘How secure do you feel your present job is? Very secure, Secure, Not very secure, Very insecure’ [32], which was dichotomised into secure vs insecure for analysis.

### Socio-demographic and health covariates

Social position in adulthood was based on measures of housing tenure and occupational Registrar General Social Class at 45 years. Housing tenure classified people according to whether they owned their housing, or lived in public housing, private rental housing or other residential arrangements. Housing tenure was recoded as ‘owner/mortgage’ versus ‘renting’ and all other categories. Registrar General Social Class was classified as non-manual (RGSC groups I, II, III non manual) and manual (RGSC groups III manual, IV, V). Marital status at 45 years was classified as married/remarried, single and separated/divorced/ widowed. Educational attainment at 33 years was grouped into three hierarchical categories: no formal educational qualifications; ‘O’ levels (lower secondary education); and ‘A’ levels or higher (higher secondary education).

### Occupational outcomes

Occupational outcomes were based on self-reported employment status at age 50 years. Leaving work at 50 years was classified as being unemployed and seeking work, being permanently sick or disabled, being wholly retired, or ‘other’ which included looking after home and family, being in full time education or ‘something else’. The reference category included employees continuing in full time or part time work and the self-employed. The ‘temporarily sick’ group which was small (n = <30) was included with the ‘permanently sick or disabled group’. The ‘permanently sick and disabled group’ also includes those on incapacity benefit.

### Missing data

Table 1 shows the prevalence of missing data within our cohort. We explored the missing data patterns and found no evidence against the assumption that any of the data were “Missing At Random” (MAR) [33]. We imputed the data under an MAR assumption through multiple imputation using chained equations (Stata ICE package). The imputation model was chosen to be congenial [34] with the most saturated model of interest. We used 50 cycles of the chained equation algorithm to create each of the 25 imputed data sets. Chains were checked for convergence.

**Table 1:** Description of working sample at 45 years and percentage of missing observations.

	% Missing		N
**Employment status at 50**	0.0%	Employed	6167
		Retired	35
		Sick	71
		Unemployed	94
		Other	98
**Sex**	0.0%	Male	3296
		Female	3214
**Housing tenure at 45**	12.2%	Owned/mortgaged	5151
		Rented	565
**Socioeconomic position at 45**	0.3%	I & II & IIInm	4539
		IIIm, IV & V nmh	1950
**Marital status at 45**	8.6%	Married or Remarried	4531
		Single never married	568
		Separated/Divorced/Widowed verses	849
**Education at 33**	10.7%	None	264
		O Level	2538
		A level or higher	3015
**Job strain**	12.5%	Low	5113
		High	586
**Job demand**	12.2%	Low	1606
		Medium	1671
		High	2436
**Decision latitude**	12.3%	Low	1983
		Medium	1857
		High	1872
**Work social support**	18.9%	Low	1909
		Medium	1821
		High	1548
**Job Security at 42**	14.3%	Very Insecure/Insecure	4773
		Secure/Very secure	809

### Statistical analysis

All data analysis was performed in Stata Version 14 (StataCorp, 2015). Unadjusted and adjusted multinomial regression models were fitted to the data to examine associations between participant’s mid-life psychosocial work environment and employment status at 50 years. These regression analyses compared the relative risks for being retired, permanently sick, unemployed and ‘other’ with the employed reference group. The adjusted models were built in a hierarchical fashion, adjusting for sex, housing tenure, socioeconomic position, marital status and education. Interactions of two mid-life psychosocial work environment measures (job strain and job insecurity) with sex, and socioeconomic position were examined; analyses were stratified when the interaction term was significant (p≤0.05).

## Results

Sociodemographic characteristics of the working sample at 45 years (n = 6510) and proportion of missing data are reported in Table 1. At age 50, 298 (4.6%) participants had stopped working whereas 6,167 continued working. At 45 years the sample was predominantly married and working in non-manual occupations with an equal sex distribution. In the working sample at age 45 years 10.3% reported high job strain, 42.6% high job demands, 34.7% low decision latitude, 33.3% low work social support and 85.5% low job security.

### Work characteristics, sociodemographic factors and work outcomes at age 50

In unadjusted analyses low and medium levels of decision latitude, low work support and high job insecurity at 45 years were associated with being unemployed at age 50 years Table 2. Conversely, having high job demands was associated with a lower risk of being unemployed at age 50 years. High job strain was associated with increased risk of being permanently sick at 50 years.

**Table 2:** Relative risks of work outcomes at age 50 for midlife sociodemographic factors and psychosocial work characteristics: Unadjusted models on imputed data. Paid work is the reference group.

		Employment status Age 50			
		Retired: RR(95%)	Sick: RR(95%)	Unemployed: RR(95%)	Other: RR(95%)
**High Job Strain**^a^		1.00 (0.29–3.52)	2.17* (1.07–4.38)	0.82 (0.36–1.87)	0.96 (0.44–2.07)
**Demand**^b^	Medium	0.82 (0.30–2.20)	0.78 (0.36–1.68)	0.60 (0.35–1.03)	0.75 (0.47–1.21)
	High	1.27 (0.54–3.01)	1.52 (0.79–2.91)	0.51* (0.29–0.87)	0.66 (0.41–1.05)
**Latitude**^c^	Low	0.99 (0.35–2.79)	1.32 (0.72–2.43)	2.04* (1.12–3.70)	1.45 (0.91–2.31)
	Medium	2.19 (0.95–5.05)	0.86 (0.44–1.68)	2.01* (1.12–3.62)	1.21 (0.75–1.95)
**Support**^d^	Low	0.64 (0.25–1.66)	1.12 (0.59–2.12)	2.18** (1.25–3.79)	0.79 (0.50–1.24)
	Medium	1.04 (0.46–2.34)	1.16 (0.60–2.21)	1.42 (0.74–2.72)	0.89 (0.54–1.46)
**Feel very insecure/ insecure with job at 42**^e^		0.26 (0.03–2.01)	0.82 (0.38–1.78)	2.41*** (1.50–3.86)	0.96 (0.55–1.66)
**Female**		1.25 (0.64–2.43)	2.06** (1.25–3.37)	0.59* (0.38–0.89)	3.58*** (2.36–5.44)
**Renting house at 45**^f^		0.57 (0.14–2.40)	2.75** (1.47–5.13)	1.64 (0.91–2.96)	0.93 (0.49–1.78)
**Socioeconomic position at 45**^g^	Manual	0.39 (0.15–1.01)	1.73* (1.07–2.78)	0.90 (0.57–1.41)	1.23 (0.85–1.77)
**Marital status at 45**^h^	Single	0.85 (0.21–3.40)	1.59 (0.75–3.35)	1.31 (0.66–2.62)	0.91 (0.47–1.76)
	Sep/W/D	1.90 (0.84–4.27)	1.95* (1.07–3.57)	1.48 (0.85–2.58)	1.07 (0.64–1.79)
**Education at 33**^i^	O level	0.95 (0.22–4.14)	0.68 (0.28–1.63)	0.62 (0.28–1.37)	0.79 (0.37–1.67)
	A level or higher	0.61 (0.14–2.69)	0.32* (0.13–0.79)	0.35** (0.16–0.78)	0.50 (0.23–1.07)

a: vs low job strain

b: 1 = medium demand 2 = high demand vs low demand

c: 1 = low latitude 2 = medium latitude vs high latitude

d: 1 = low support, 2 = medium support vs high support

e: vs secure/very secure

f: vs owned/mortgaged

g: 1 = IIIm & IV & V nmh vs I & II & IIInm

h: 1 = single never married, 2 = separated/divorced/widowed vs married or remarried

i: 1 = O level, 2 = A level or higher vs none

p < * 0.05, **0.01, ***0.001

### Adjusted analyses of work characteristics and work outcomes at age 50

After adjustment for sex, housing tenure, socioeconomic status, marital status and education, high demands were associated with an increased risk of being permanently sick at age 50 Table 3. Low and medium levels of decision latitude and low work support were all associated with an increased risk of being unemployed at age 50. High levels of demand were associated with a lower risk of being unemployed at age 50. High job insecurity was associated with increased risk of being unemployed at age 50 years Table 3.

**Table 3:** Relative risks of work outcomes at age 50 for psychosocial work characteristics: Adjusted models on imputed data (sex, housing tenure, socioeconomic, marital status, education). Paid work is the reference group.

		Employment status Age 50			
		Retired: RR(95%)	Sick: RR(95%)	Unemployed: RR(95%)	Other: RR(95%)
**High Job Strain**^a^		0.91 (0.25–3.27)	1.97 (0.97–4.01)	0.78 (0.34–1.77)	0.87 (0.40–1.86)
**Demand**^b^	Med	0.77 (0.28–2.09)	0.93 (0.43–2.03)	0.58 (0.33–1.00)	0.86 (0.53–1.38)
	High	1.16 (0.48–2.84)	2.14* (1.09–4.21)	0.50* (0.29–0.88)	0.77 (0.47–1.27)
**Latitude**^c^	Low	0.97 (0.33–2.91)	0.86 (0.45–1.65)	2.01* (1.06–3.79)	1.06 (0.65–1.73)
	Med	2.15 (0.92–5.02)	0.71 (0.36–1.40)	2.05* (1.13–3.71)	1.04 (0.64–1.68)
**Support**^d^	Low	0.66 (0.25–1.76)	1.32 (0.68–2.56)	1.96* (1.12–3.44)	1.02 (0.64–1.64)
	Med	1.04 (0.46–2.35)	1.25 (0.65–2.40)	1.36 (0.71–2.61)	0.99 (0.60–1.63)
**Feel very insecure/ insecure with job at 42**^e^		0.27 (0.04–2.08)	0.88 (0.40–1.92)	2.27*** (1.41–3.67)	1.12 (0.64–1.95)

a: vs low job strain

b: 1 = medium demand 2 = high demand vs low demand

c: 1 = low latitude 2 = medium latitude vs high latitude

d: 1 = low support, 2 = medium support vs high support

e: vs secure/very secure.

p < * 0.05, **0.01, ***0.001

We found no interactions of social class (non-manual vs manual) and sex with job strain and job insecurity in their association with work outcomes at age 50.

## Discussion

### Summary of findings

In fully adjusted analyses low decision latitude, low work support and high job insecurity were associated with increased risk of being unemployed at 50 years. Medium and high demands were associated with reduced risk of being unemployed at 50 years. High demands were associated with increased risk of permanent sickness at 50 years. In fully adjusted analyses work characteristics were not associated with retirement at 50 years.

### Interpretation of results

Insecure jobs of low psychosocial quality at 45 years are associated with unemployment at 50 years. By their nature these are likely to be temporary, and high job insecurity may signal this. Unemployment at 33 years has been related to manual social class of origin in this cohort [35]. Nevertheless, of those unemployed at 50 years, 55% have education at ‘A’ levels or above and 70% are women. At 45 years 48% of these women were in RGSC I and II, and 22% in IV and V so this association is not confined to manual occupations. Early exit from the labour force is increased by high general levels of unemployment which may be exacerbated by this survey being undertaken at the start of the economic recession in 2008. Those unemployed at 50 years may have left because of low psychosocial quality working conditions [3] or may have been made redundant. Employers may be less supportive of older workers which may lead to older employees, sensitive to employers and colleagues view of them, leaving employment, and because they see a lack of future prospects [36]. Once unemployed it is more challenging to return to the workforce after the age of 50 years than at earlier ages [37,38] although in this cohort two thirds of those unemployed at 50 years were re-employed by the age 55 years. High demands, which in earlier studies, represented higher status jobs, in which employees are ‘in demand’ are associated with reduced odds of unemployment [24].

The association of high levels of demands with increased risk of permanent sickness may reflect a causal association mediated through adulthood psychiatric disorder. It may also be that the combination of work demands and psychiatric disorder have a synergistic effect on permanent sickness–the association of demands with permanent sickness was no longer significant after adjustment for psychiatric disorder measured by the malaise scale at either 23, 33 or 42 years (results not shown)[26, 39]]. Less advantaged social position was also strongly associated with permanent sickness at 50 years. The lack of association between work characteristics and retirement may be because at 50 years this cohort had largely not yet considered retirement having not reached the statutory pension age of 62 years for women and 65 years for men unlike many other studies [1–3, 5, 9–11].

### Comparison to other studies

The association of low decision latitude with unemployment is found in other studies [2,24, 40–42]]. As in other studies we found that high job strain and low decision latitude predicts increased risk of permanent sickness, measured in other studies as being on disability benefits [17–19,27,43]. We did find that high levels of demands predicted increased risk of permanent sickness, as in other studies, in which permanent sickness was measured by disability pension [20,21]. It is interesting that high demands were associated with both reduced risk of unemployment and increased risk of permanent sickness. This may be due to the measurement of self-reported demands. In Samuelsson et al’s study objectively measured demands derived from a job exposure matrix were related to increased risk of disability pension for psychiatric disorders [21]. These analyses carried out in a twin sample were able to rule out the effect of familial factors (e.g. shared social status) on these associations. This study, which supports our findings, seems to be strong evidence for an effect of demands on increasing the risk of permanent sickness. On the other hand, our association between high demands and reduced unemployment may be because our demands questions are partly measuring ‘challenge stressors’ which have been consistently associated with labour force retention rather than ‘hindrance stressors’ which are more like the conventional definition of demands and are related to decreased job satisfaction and increased job turnover [20, 44].

Almost by definition job insecurity predicts unemployment [45] and is seen as ‘the most extreme stage of job instability’ [46] and this may be especially the case for older people at a time of economic recession. Less advantaged socioeconomic status is also a risk factor for unemployment in other studies [24]. In the United Kingdom one million people aged 50–64 years have been estimated to have left the workforce involuntarily between 2006 and 2014 and 26% of those currently jobless in this age group would like to be working [37]. Of the unemployed population over 50 years in the UK, 46.6% have been out of work for 12 months or more. Unemployment rates in this age group tend to be higher among women, as we found too. Unemployment at age 50 may mean restricted opportunities for re-employment in many occupations. There may also be an effect of the ‘discouraged workers’ concept where people perceive that it is no longer worth looking for work which could be influenced by previous experience of working conditions.

### Strengths and limitations

The study limitations include the relatively brief measures of work characteristics at age 45 years, at a single wave of data collection, and a lack of detail on the work outcomes provided by self-report at 50 years. The cohort structure of the sample means that there is no variation in period of exposure but the cohort structure is also a strength as it provides longitudinal risk factor and health data from across the lifecourse in a large sample undergoing work transitions at age 50 years which is not confounded by age. Due to attrition across the lifecourse the sample is no longer as representative of the general population and may have lost some socially disadvantaged participants., Employment rates in this cohort were comparable with contemporary national data. Having only small numbers of employees at 45 years who retired at the age of 50 years was also a limitation.

## Conclusions

Adverse psychosocial working conditions and job insecurity at 45 years predict unemployment by age 50 years. High job demands are associated with permanent sickness at 50 years; this may be accounted for by an association with adulthood psychological distress. Early exit from the workforce may lead to financial difficulties in old age because of lack of pension. Improvements to psychosocial working conditions in midlife may help to retain employees beyond the age of 50 years in the workforce through reducing unemployment. Employers should be more open to valuing older worker’s skills and experience [37].
